# Development and validation of the HCV-MOSAIC risk score to assist testing for acute hepatitis C virus (HCV) infection in HIV-infected men who have sex with men (MSM)

**DOI:** 10.2807/1560-7917.ES.2017.22.21.30540

**Published:** 2017-05-25

**Authors:** Astrid M Newsum, Ineke G Stolte, Jan TM van der Meer, Janke Schinkel, Marc van der Valk, Joost W Vanhommerig, Anne Buvé, Mark Danta, Arjan Hogewoning, Maria Prins, on behalf of the MOSAIC (MSM Observational Study of Acute Infection with hepatitis C) study group

**Affiliations:** 1 Department of Infectious Diseases Research and Prevention, Public Health Service of Amsterdam, Amsterdam, the Netherlands; 2 Department of Internal Medicine, Center for Infection and Immunity Amsterdam (CINIMA), Academic Medical Center, Amsterdam, the Netherlands; 3 Department of Medical Microbiology, Academic Medical Center, Amsterdam, the Netherlands; 4 Department of Public Health, Institute of Tropical Medicine, Antwerp, Belgium; 5 St Vincent’s Clinical School, University of New South Wales, Sydney, Australia; 6 STI Outpatient Clinic, Public Health Service of Amsterdam, Amsterdam, the Netherlands; 7 The members of the group are listed at the end of the article

**Keywords:** hepatitis C, diagnosis, clinical prediction rule, risk score, MSM, testing

## Abstract

Current guidelines recommend hepatitis C virus (HCV) testing for HIV-infected men who have sex with men (MSM) with ongoing risk behaviour, without specifying the type of risk behaviour. We developed and validated the HCV-MOSAIC risk score to assist HCV testing in HIV-infected MSM. The risk score consisted of six self-reported risk factors identified using multivariable logistic regression using data from the Dutch MOSAIC study (n = 213, 2009–2013). Area under the ROC curve (AUC), sensitivity, specificity, post-test-probability-of-disease and diagnostic gain were calculated. The risk score was validated in case–control studies from Belgium (n = 142, 2010–2013) and the United Kingdom (n = 190, 2003–2005) and in cross-sectional surveys at a Dutch sexually transmitted infections clinic (n = 284, 2007–2009). The AUC was 0.82; sensitivity 78.0% and specificity 78.6%. In the validation studies sensitivity ranged from 73.1% to 100% and specificity from 56.2% to 65.6%. The post-test-probability-of-disease ranged from 5.9% to 20.0% given acute HCV prevalence of 1.7% to 6.4%, yielding a diagnostic gain of 4.2% to 13.6%. The HCV-MOSAIC risk score can successfully identify HIV-infected MSM at risk for acute HCV infection. It could be a promising tool to improve HCV testing strategies in various settings.

## Introduction

Studies on hepatitis C virus (HCV) infections among HIV-infected men who have sex with men (MSM) have provided insights into the epidemiology and risk factors for sexually transmitted HCV acquisition [1,2]. As HCV transmission among MSM is ongoing in high-income countries worldwide [3,4], targeted testing is needed. Current national and international clinical guidelines recommend at least annual HCV antibodies (anti-HCV) testing for HIV-infected MSM who have unprotected (condomless) sex or who have been exposed to other, unspecified risk factors [5-7]. Furthermore, bi-annual alanine aminotransferase (ALT) testing is recommended for all HIV-infected patients [6,7]. In case of unexplained elevated ALT levels, subsequent HCV-RNA testing can be performed at the discretion of the physician. However, ALT is often not routinely measured in sexually transmitted infection (STI) clinics or other places outside of HIV care. Also, anti-HCV testing might not be sufficient in cases of an acute HCV infection as it takes several weeks or even months before anti-HCV can be detected in the presence of HIV [8,9]. Moreover, these guidelines include the presence of risk behaviour without specifying type and frequency.

Since early HCV detection and treatment may prevent onward transmission [10], more specific recommendations are required to identify who should be tested for acute HCV. A risk questionnaire could reduce the number of HCV tests performed in HIV-infected MSM, lowering costs and enhancing implementation of acute HCV testing in, for example, STI clinics. For chronic HCV infections, several risk scores or screening strategies to target those at highest risk for HCV were developed [11-16]. However, to the best of our knowledge, risk scores identifying MSM at increased risk for acute HCV infection do not exist.

Recently, we examined risk factors for acute HCV infection in the MOSAIC study (MSM Observational Study of Acute Infection with hepatitis C). The MOSAIC study is an ongoing, prospective, observational cohort, enrolling HIV-infected MSM with acute HCV infection (cases) and one or two controls without a history of HCV for each case [17]. In this study we found that a high number (four or more) of risky sex acts was strongly associated with HCV acquisition [18]. Therefore, in the present study, we developed a risk score identifying at-risk MSM using data from this MOSAIC study and evaluated its sensitivity and specificity. In addition, we evaluated the performance of this risk score in three different populations of HIV-infected MSM, to assess whether this tool could be used to assist testing for acute HCV infection in HIV-infected MSM.

## Methods

### Development of the risk score

For the development of the risk score, all cases and controls enrolled in the MOSAIC study before February 2014 were selected. Acute HCV infection was defined as an interval ≤ 6 months between the first positive HCV-RNA test and the preceding negative HCV-RNA or anti-HCV test. Information on risk factors for HCV was obtained using a detailed self-administered questionnaire. Questions about risk behaviour refer to the 6 months preceding the moment of diagnosis with acute HCV for cases, and the 6 months preceding study entry for controls, except for questions about drug use and STIs, which refer to the past 12 months. The MOSAIC study was approved by the Institutional Review Board of the Academic Medical Center at the University of Amsterdam and ethical committees/board of directors of each institute recruiting participants; the assigned study numbers are NL26485.018.09 and NL48572.018.14.

For the development of the original risk score, we selected all risk factors that were statistically significantly associated with acute HCV in the multivariable logistic regression model including variables that potentially have direct effects on acquisition and variables that potentially facilitate transmission of acute HCV, as described elsewhere [18]. Subsequently, an individual risk score for each patient was calculated by summing the logistic regression beta-coefficients of all significant (p value < 0.05) risk factors reported.

Since the questions in the MOSAIC questionnaire are very detailed, we adjusted the original risk score to a revised risk score, which we will refer to as the HCV-MOSAIC risk score. For the HCV-MOSAIC risk score we used simplified definitions of the risk factors identified for the original risk score, making it suitable for validation and implementation. The HCV-MOSAIC risk score was constructed using the different beta coefficients derived from multivariable logistic regression analysis entering these simplified variables.

### Validation of the risk score

We validated the HCV-MOSAIC risk score using three different study populations, for which we obtained the primary datasets. The first was a case–control study among HIV-infected MSM in care in three AIDS Reference Centers in Belgium from 2010 until 2013 [19]. Screening for anti-HCV was performed, followed by confirmation of positive samples by detection of HCV-RNA. All included participants had a negative anti-HCV test during the 12 months before their positive HCV test. For each case, the first two HIV-infected anti-HCV-negative MSM who visited the clinic after the case was included were selected as controls. The second was a case–control study in HIV clinics in the United Kingdom (UK) from 2003 until 2005 [20]. Cases were HIV-infected MSM with acute HCV infection, defined as a documented seroconversion to anti-HCV, accompanied by a positive HCV-RNA and/or clinical and biochemical criteria. The aim was to match two MSM controls without HCV for age, length of HIV infection, ethnicity and combination antiretroviral therapy (cART) exposure status. The third cohort was based on anonymous bi-annual cross-sectional surveys conducted at the STI clinic of the Public Health Service of Amsterdam in the Netherlands [21]. We used data collected between 2007 and 2009. Anti-HCV and HCV-RNA testing were performed in all HIV-infected MSM. Acute/recent HCV infection was defined as (i) HCV-RNA-positive and anti-HCV-negative or (ii) HCV-RNA-positive and anti-HCV-positive without a self-reported history of a previous positive HCV test. All other MSM with both a positive HCV-RNA and anti-HCV were excluded from the STI clinic dataset. The MSM who did not fulfil the criteria for acute/recent HCV infection were included in the analysis as HCV-negative.

Risk factors for HCV were collected at interview using a standardised questionnaire [19,21] or by a self-administered questionnaire [20]. Questions about risk behaviour referred to the 12 months before HCV diagnosis or study entry in the two case–control studies, and to the previous 12 months in the cross-sectional surveys.

### Statistical analysis

Using the MOSAIC data, the optimal cut-off point of the risk score to predict HCV positivity, defined as the highest sensitivity in combination with the highest specificity, was determined using Receiver Operating Characteristic (ROC) curves. The area under the curve (AUC) was calculated to assess accuracy of the risk score. Sensitivity and specificity with Wilson Score 95% confidence intervals (CI) were calculated for the optimal cut-off point. Differences between sensitivity and specificity from the development study and validation studies were evaluated using Newcombe’s method 10 for independent proportions [22]. If the answer to a risk factor question was missing for a patient, we assumed that this risk factor was not present.

We could not reliably determine the positive and negative predictive value of the risk score, as these measures are dependent on the infection prevalence in the study group, and the case–control distribution in the development and validation studies, except for the Dutch STI clinic surveys, does not reflect the actual prevalence of acute HCV. To assess the clinical relevance, we calculated the post-test probability of HCV infection (i.e. the likelihood of being HCV-positive when given a positive HCV testing advice based on the risk score) using the formula [23]:

$$\frac{\left(sensitivity\;\times \;prevalence\right)}{sensitivity\;\times \;prevalence\;+\;\left(1\;-\;specificity\right)\;\times \;(1\;-\;prevalence)}$$

As the post-test probability of infection depends largely on the pre-test probability of infection (i.e. the prevalence of acute HCV infection in HIV-infected MSM, which we calculated using the data from the Dutch STI clinic surveys with its 95% CI as range), Fagan’s nomogram [24] was used to visualise the diagnostic gain (post-test probability minus pre-test probability of infection) after a positive testing advice. This graphical calculation of Bayes’ theorem describes how positive testing advice changes the infection probability by combining the pre-test probability of acute HCV infection with the likelihood ratio (LR) of the risk score (which is calculated from sensitivity and specificity [23]), resulting in the post-test probability of acute HCV infection. All analyses were performed using Stata version 13.1 (Stata Statistical Software: Release 13; StataCorp LP, College Station, Texas, US).

## Results

The MOSAIC development study enrolled 82 HIV-infected MSM with acute HCV and 131 HIV-infected MSM without a history of HCV as controls. The first validation study from Belgium included 52 cases and 90 controls and the second from the UK, 60 cases and 130 controls. Third, we included 10 HIV-infected MSM with acute HCV and 274 without HCV from the Dutch STI clinic surveys. Characteristics of the development and validation studies and their study populations are shown in Table 1; the median age of participants in all validation studies was significantly lower than the median age in the development study (p value < 0.05 for all studies, Mann–Whitney U-test).

**Table 1:** Characteristics of the development and three validation studies and their study populations and the variables of the HCV-MOSAIC risk score

Characteristics	Development study		Validation studies		
	**MOSAIC study, the Netherlands** **(n = 213)**		**Case–control study, Belgium** **(n = 142)**	**Case–control study, UK** **(n = 190)**	**Dutch STI clinic surveys** **(n = 284)**
**Study design**	Case–control		Case–control	Case–control	Cross-sectional
**HCV status** **- HCV-positive (n)** **- HCV-negative (n)**	82^a^ 131		52 90	60 130	10 274
**Study period**	2009–2013		2010–2013	2003–2005	2007–2009
**Median age in years (IQR)**	45.7 (41.0–52.2)		45.0 (37.0–51.0)^b^	38.0 (33.5–41.9)^c^	42.0 (35.0–47.0)
				
**Self-reported** **variables in the risk score**	**HCV-MOSAIC risk score**	**beta**	**Deviations from the HCV-MOSAIC risk score**		
**Condomless RAI 6M**	Yes / no	1.1	RAI and condomless AI asked separately	ND	ND
**Sharing of sex toys 6M**	Yes / no	1.2	With casual sex partner(s)	ND	ND
**Unprotected fisting 6M**	Yes / no	0.9	ND	ND	ND
**Injecting drug use 12M**	Yes / no	1.4	During sex	ND	ND
**Sharing of straws when NAD used 12M**	Yes / no	1.0	ND	ND	Not asked
**Ulcerative STI 12M**	Yes / no	1.4	ND	Ever had syphilis or herpes	Not self‑reported but tested

AI: anal intercourse; HCV: hepatitis C virus; IQR: interquartile range; MOSAIC: MSM (men who have sex with men) Observational Study of Acute Infection with hepatitis C; NAD: nasally administered drug; ND: no deviation; RAI: receptive anal intercourse; STI: sexually transmitted infection; ulcerative STI: syphilis, genital herpes or lymphogranuloma venereum infection; UK: United Kingdom; 6M: during the past 6 months; 12M: during the past 12 months.

^a^ Nine reinfections.

^b^ One missing value.

^c^ Twenty seven missing values.

### Development of the risk score

**Figure 1:**
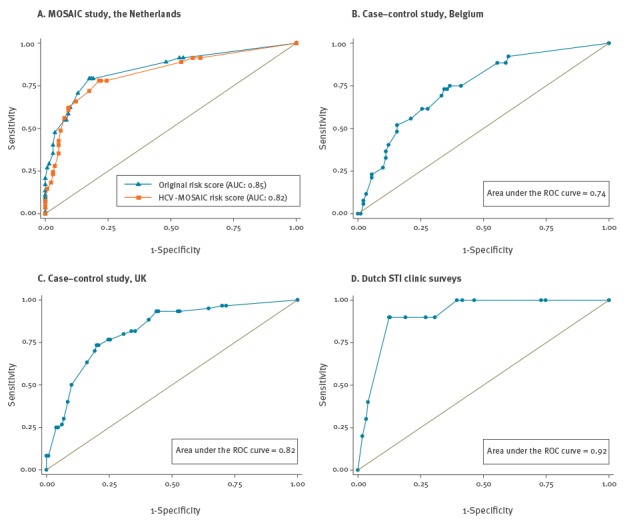
Receiver operating characteristic curves for the original and HCV-MOSAIC risk score in the development study (A) and for the HCV-MOSAIC risk score in the three validation studies (B–D)

AUC: area under the ROC curve; HCV: hepatitis C virus; MOSAIC: MSM (men who have sex with men) Observational Study of Acute Infection with hepatitis C; ROC: receiver operating characteristic; STI: sexually transmitted infection; UK: United Kingdom.

The previously described logistic regression model [18] identified the following six dichotomous risk factors for the original risk score: (i) condomless receptive anal intercourse (RAI) (beta 1.6); (ii) sharing of sex toys (beta 1.3) (both (i) and (ii) with HCV-positive or HCV-unknown sex partners); (iii) unprotected fisting (fisting without gloves, or with gloves but also group sex reported, beta 0.9); (iv) injecting drug use (IDU) during sex (beta 2.7); (v) sharing of straws when nasally administered drugs (NAD) used (beta 1.2); and (vi) self-reported ulcerative STI (syphilis, genital herpes or lymphogranuloma venereum infection, beta 1.6). Although statistically significant in the model, we excluded CD4 cell count, since its inclusion would make the risk score unusable in a setting where CD4 cell counts are not routinely measured (e.g. STI clinic). The best cut-off point for the original risk score, as determined using the ROC-curve (Figure 1A, AUC 0.85, 95% CI: 0.79–0.90) was ≥ 2.5. Sensitivity and specificity of the risk score using this cut-off point were 79.3% (95% CI: 69.3–86.6) and 82.4% (95% CI: 75.0–88.0) respectively.

For development of the HCV-MOSAIC risk score, as described in the methods we simplified the first four of the six risk factors, resulting in the following risk factors: (i) condomless RAI (with any partner, beta 1.1); (ii) sharing of sex toys (with any partner, beta 1.2); (iii) unprotected fisting (fisting without gloves, beta 0.9); (iv) IDU in the past 12 months (beta 1.4); (v) sharing of straws when NAD used (beta 1.0); and (vi) ulcerative STI (beta 1.4) (Table 1). The optimal cut-off point for the HCV-MOSAIC risk score became ≥ 2.0 and the ROC-curve had an AUC of 0.82 (95% CI: 0.76–0.88) (Figure 1A). When compared with the original risk score, the sensitivity of the HCV-MOSAIC risk score slightly dropped from 79.3% to 78.0% (95% CI: 67.9–85.6) and the specificity from 82.4% to 78.6% (95% CI: 70.8–84.8). The proportion of all participants with a risk score of ≥ 2.0 was 43% (92/213).

### Validation of the risk score

The sensitivity and specificity of the HCV-MOSAIC risk score in the Belgian case–control study were 73.1% (95% CI: 59.7–83.2) and 65.6% (95% CI: 55.3–74.6), respectively. In the case–control study from the UK, sensitivity and specificity were 93.3% (95% CI: 84.1–97.4) and 56.2% (95% CI: 47.6–64.4), respectively. In the Dutch STI clinic surveys, sensitivity and specificity were 100% (95% CI: 72.2–100) and 60.6% (95% CI: 54.7–66.2), respectively (Table 2).

**Table 2:** Performance of the HCV-MOSAIC risk score among HIV-infected men who have sex with men in the development and three validation studies

	Development study	Validation studies		
	MOSAIC study, the Netherlands	Case–control study, Belgium	Case–control study, UK	Dutch STI clinic surveys
**Sensitivity** **(95% CI)**	78.0% (67.9–85.6)	73.1% (59.7–83.2)	93.3% (84.1–97.4)	100% (72.2–100)
**Specificity** **(95% CI)**	78.6% (70.8–84.8)	65.6% (55.3–74.6)	56.2% (47.6–64.4)	60.6% (54.7–66.2)
**Proportion to be tested^a^**	43%	49%	59%	42%
**Area under the** **ROC curve** **(95% CI)**	0.82 (0.76–0.88)	0.74 (0.66–0.83)	0.82 (0.76–0.88)	0.92 (0.85–0.98)

CI: confidence intervals; HCV: hepatitis C virus; MOSAIC: MSM (men who have sex with men) Observational Study of Acute Infection with hepatitis C; ROC: receiver operating characteristic; STI: sexually transmitted infection; UK: United Kingdom.

^a^ Proportion of all cases and controls with a risk score of ≥ 2.0.

In the Belgian case–control study and the Dutch STI clinic surveys the sensitivity was lower and higher respectively than in the development study, but these differences were not statistically significant. In the study from the UK the sensitivity was significantly higher than in the development study (difference 15.3%, 95% CI: 3.3–26.2). Specificity was significantly lower in all validation studies compared with the development study (difference for the Belgian study 13.0%, 95% CI: 1.2–25.0, the UK study 22.4%, 95% CI: 11.1–33.0, and the Dutch study 18.0%, 95% CI: 8.5–26.6). The AUC in the validation studies ranged from 0.74 to 0.92 (Figure 1B–D). The proportion of participants (both cases and controls) with a risk score of ≥ 2.0 (i.e. the proportion of the population to be tested) in the validation studies ranged from 42% to 59% (Table 2). Table 3 shows the performance of the HCV-MOSAIC risk score for a variety of cut-offs in both the development and validation studies.

**Table 3:** Performance of the HCV-MOSAIC risk score for a range of different cut-offs among HIV-infected men who have sex with men in the development and three validation studies

	Development study		Validation studies					
	MOSAIC study, the Netherlands		Case–control study, Belgium		Case–control study, UK		Dutch STI clinic surveys	
**Cut‑off^a^**	**Sensitivity (%)**	**Specificity (%)**	**Sensitivity (%)**	**Specificity (%)**	**Sensitivity (%)**	**Specificity (%)**	**Sensitivity (%)**	**Specificity (%)**
**≥ 0.9**	91.5	38.2	92.3	40.0	96.7	28.5	100.0	25.2
**≥ 1.1**	89.0	45.8	88.5	44.4	95.0	35.4	100.0	27.0
**≥ 1.4**	78.1	75.6	75.0	63.3	93.3	47.7	100.0	58.4
**≥ 2.0**	78.1	78.6	73.1	65.6	93.3	56.2	100.0	60.6
**≥ 2.1**	72.0	82.4	69.2	66.7	88.3	59.2	90.0	69.3
**≥ 2.3**	65.9	87.8	61.5	74.4	81.7	64.6	90.0	73.0
**≥ 2.5**	61.0	90.8	55.8	78.9	80.0	69.2	90.0	81.0
**≥ 3.2**	48.8	93.9	48.1	84.4	73.3	79.2	90.0	87.6
**≥ 3.4**	40.2	94.7	36.5	88.9	70.0	80.8	40.0	96.0
**≥ 4.6**	14.6	99.2	21.2	94.4	30.0	93.1	20.0	98.2

HCV: hepatitis C virus; MOSAIC: MSM (men who have sex with men) Observational Study of Acute Infection with hepatitis C; STI: sexually transmitted infection; UK: United Kingdom.

^a^ Results are shown only for the cut-offs that were available in all four studies.

In the Dutch STI clinic surveys, data on one of the variables in the risk score (sharing of straws when NAD used) were not collected and therefore not scored. In a sensitivity analysis, we restricted the HCV-MOSAIC risk score in the development study to the same risk factors measured in the STI clinic (i.e. excluding sharing of straws): sensitivity decreased from 78.0% to 70.7% (95% CI: 60.1–79.5) and specificity increased from 78.6% to 83.2% (95% CI: 75.9–88.6).

### Post-test probability

**Figure 2:**
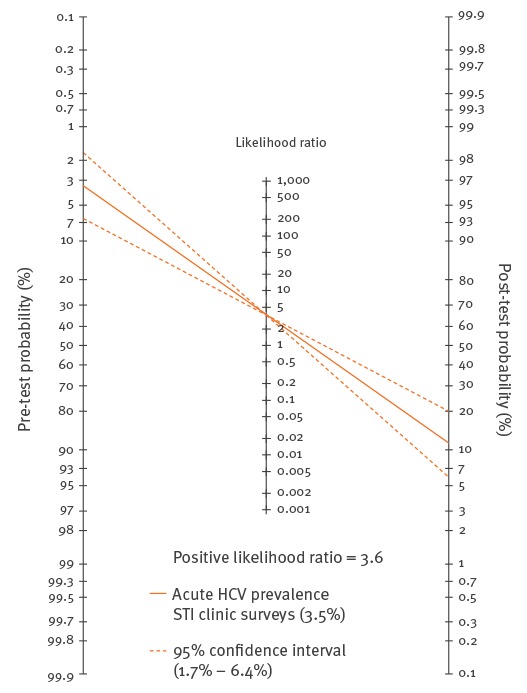
Fagan’s nomogram for a risk score of ≥ 2.0

HCV: hepatitis C virus; MOSAIC: MSM (Men who have sex with men) Observational Study of Acute Infection with hepatitis C; STI: sexually transmitted infection.

The post-test probability of acute HCV infection was calculated using the sensitivity and specificity of the HCV-MOSAIC risk score in the development study and using the prevalence of acute HCV in HIV-infected MSM in the Dutch STI clinic surveys, which was 3.5% (10/284 MSM, 95% CI: 1.7–6.4). The Fagan’s nomogram (Figure 2) shows the post-test probability for a risk score of ≥ 2.0 and gives a precise overview of diagnostic gain.

The Fagan’s nomogram combines a range of pre-test probabilities of acute hepatitis C virus (HCV) infection (i.e. the prevalence range) with the likelihood ratio (LR) of the risk score, resulting in a range of post-test probabilities of acute HCV infection. It visualises diagnostic gain of the risk score (i.e. post-test probability minus pre-test probability).

The lines that start at the left y-axis show the HCV pre-test probability (i.e. 3.5%, range 1.7–6.4), cross the LR for a risk score of ≥ 2.0 (positive LR, i.e. sensitivity/(1–specificity)), then point to the HCV post-test probability at the right y-axis, which is 11.7% (range 5.9–20.0). The diagnostic gain of the risk score equals the difference between the infection probability for an individual before filling out the risk score (i.e. the prevalence) and the infection probability for an individual after being assigned to undergo HCV testing according to the risk score (i.e. HCV post-test probability). The diagnostic gain was 8.2% (11.7% minus 3.5%) and varied from 4.2% (5.9% minus 1.7%) to 13.6% (20.0% minus 6.4%).

## Discussion

We developed and validated the first risk score for acute HCV infection in HIV-infected MSM. Using this risk score, 42–59% of HIV-infected MSM would be advised to undergo HCV testing, correctly identifying 73–100% of HIV-infected MSM with acute HCV infection, potentially making it a useful tool to assist testing for acute HCV infection. Our risk score could be implemented in settings where HIV-infected MSM are being tested for STIs, e.g. STI clinics. Currently, HCV testing is not routinely offered to MSM attending STI clinics in the Netherlands [25]. Moreover, the risk score could be an addition to the current guidelines for HCV testing where risk behaviour as test criterion is not specified. Since all questions are self-reported, the development of a mobile-compatible website or application containing the risk score could be practical, ensuring confidentiality.

Although we consistently found > 70% sensitivity, we need to emphasise that there is a proportion of HIV-infected MSM with acute HCV infection that will be missed when using the risk score. As described above this risk score should therefore be used as an additional tool rather than a replacement of testing practices in HIV clinics. Also, since the specificity was around 60% in the validation studies, a substantial proportion of HCV-negative MSM will be falsely identified as possible HCV-positive. However, since these MSM have a high score, our risk score could also be used to identify those who would benefit from interventions to reduce risk behaviour to prevent HCV infection.

Sensitivity and specificity of our risk score are within the higher range of those reported for existing risk scores to detect chronic HCV infection [11-16] and are also favourably comparable to existing risk scores to predict early HIV infection [26-28]. The diagnostic gain of the risk score ranged from 4.2% to 13.6%, which is slightly higher compared with the diagnostic gain of a risk assessment questionnaire for chronic HCV infection in the general population [14]. However, the diagnostic gain is dependent on the acute HCV prevalence in the population in which the risk score will be used and increases when prevalence is higher. A recent systematic review estimated a prevalence range of active HCV infection in HIV-infected MSM of 5.3–7.3% [29]. This range includes the upper limit of the prevalence we used (i.e. 6.4%). Use of our risk score will result in 42–59% of a population to be tested for HCV instead of everyone, which could potentially reduce test costs. However, cost-effectiveness studies are needed to compare different HCV testing strategies.

Our study has several limitations. First, there is heterogeneity between the development and validation studies. The performance of the risk score may have been influenced by differences in the definition of acute HCV between studies. We found 100% sensitivity in the Dutch STI clinic surveys, where it is likely that none of the acute HCV cases were missed because all men were simultaneously tested for HCV-RNA and anti-HCV. In addition, the questionnaires in the validation studies referred to risk behaviour in the last 12 months, whereas 3 of the 6 risk factors in our risk score refer to the last 6 months. The longer time period could have led to more risk behaviour acts reported, leading to a higher proportion with a risk score of ≥ 2.0. Also, study periods, countries and mode of questionnaire (at interview or self-administered) differed, and changes in risk behaviour over time or the social acceptability of some of the answers could have affected the performance of the risk score. Differences in HCV prevalence over time and between regions may have resulted in differences in the chance for an individual of being exposed to HCV, regardless of the level of risk behaviour. Second, we were unable to take into account the predictive value of an elevated ALT, since for the majority of the MOSAIC cases, HCV testing and diagnosis were based on an elevated ALT level. As current HCV testing practices in HIV treatment centres largely rely on the presence of an elevated ALT, the additional value of our risk score in combination with an elevated ALT can only be measured using a prospective validation study, as this would require testing for acute HCV in all patients with and without elevated ALT. We believe our risk score can be of added value, as ALT levels may remain within normal limits or rapidly normalise after acute HCV infection [30,31] and the sensitivity of an elevated ALT is reported to be as low as 20% for a recent HCV infection [31]. Third, our risk score was developed using data from a case–control study, while preferably a risk score should be developed using a prospective cohort study of HIV-infected MSM who are being regularly tested for acute HCV infection. A fourth limitation is that the sample sizes of the development and validation studies were relatively small.

Our risk score has not been validated among HIV-negative MSM, as their HCV prevalence is relatively low [21,32]. However, HCV infections have been reported in HIV-negative MSM using HIV pre-exposure prophylaxis [33-35]. For those people, it would be worth evaluating whether the risk score could assist HCV testing. Furthermore, our risk score was neither primarily developed nor validated for HCV reinfections. As reinfections are reported to be common in MSM [30,36,37], it could also be useful to validate the HCV-MOSAIC risk score in this group.

In conclusion, the HCV-MOSAIC risk score identifies HIV-infected MSM at risk for acute HCV infection. We encourage the use of this risk score, especially at testing locations where MSM are not regularly tested for HCV or where ALT is not routinely measured. It could be a valuable addition to the current guidelines for HCV testing and potentially reduce the amount of tests performed in MSM at low risk for acute HCV infection. In addition, it could be used as a tool to identify those who would benefit from interventions to reduce risk behaviour to prevent acute HCV infection.

## Notes

**MOSAIC collaborators:** J.T.M. van der Meer, R. Molenkamp, M. Mutschelknauss, H.E. Nobel, H.W. Reesink, J. Schinkel, M. van der Valk, J.W. Vanhommerig (Academic Medical Center, Amsterdam, the Netherlands); G.E.L. van den Berk, K. Brinkman, D. Kwa, N. van der Meché, A. Toonen, D. Vos (Onze Lieve Vrouwe Gasthuis, Amsterdam, the Netherlands); M. van Broekhuizen, F.N. Lauw, J.W. Mulder (MC Slotervaart, Amsterdam, the Netherlands); J.E. Arends, A. van Kessel, I. de Kroon (University Medical Center Utrecht, Utrecht, the Netherlands); A. Boonstra, M.E. van der Ende, S. Hullegie, B.J.A. Rijnders (Erasmus Medical Center, Rotterdam, the Netherlands); T.J.W. van de Laar (Sanquin Blood Supply Foundation, Amsterdam, the Netherlands); L. Gras, C. Smit (Stichting HIV Monitoring, Amsterdam, the Netherlands); A.M. Newsum, M. Prins, W. van der Veldt (Public Health Service of Amsterdam, Amsterdam, the Netherlands).
